# Non-Pharmacological Nursing Interventions for Prevention and Treatment of Delirium in Hospitalized Adult Patients: Systematic Review of Randomized Controlled Trials

**DOI:** 10.3390/ijerph18168853

**Published:** 2021-08-22

**Authors:** Yoonyoung Lee, Jongmin Lee, Jeounghee Kim, Youngsun Jung

**Affiliations:** 1 Department of Nursing, Sunchon National University, Jungang-ro, Jeonnam, Suncheon 57922, Korea; 2 Department of Nursing, Asan Medical Center, Seoul 05505, Korea; nurjongmin@gmail.com (J.L.); jeounghee@amc.seoul.kr (J.K.); jys@amc.seoul.kr (Y.J.)

**Keywords:** delirium, hospitalization, non-pharmacologic intervention, nursing intervention, systematic review

## Abstract

Delirium is a common neurobehavioral complication in hospitalized patients that can occur in the acute phase and lead to poor long-term outcomes. The purpose of this study was to identify non-pharmacological nursing interventions for the prevention and treatment of delirium in hospitalized adult patients. We conducted a systematic review to synthesize the findings of published studies. We searched the PubMed, EMBASE, CINAHL, and Cochrane Library CENTRAL databases for randomized controlled trials in January 2021. We report this systematic review according to the PRISMA 2009 checklist. The study was registered on PROSPERO (CRD42021226538). Nine studies were systematically reviewed for non-pharmacological nursing interventions for the prevention and treatment of delirium. The types of non-pharmacological nursing interventions included multicomponent intervention, multidisciplinary care, multimedia education, music listening, mentoring of family caregivers concerning delirium management, bright light exposure, ear plugs, and interventions for simulated family presence using pre-recorded video messages. These results could help nurses select and utilize non-pharmacological nursing interventions for the prevention and treatment of delirium in clinical nursing practice.

## 1. Introduction

Delirium is a common neurobehavioral complication in hospitalized patients [1]. Delirium occurs in the acute phase and can lead to poor long-term outcomes [2]. To prevent these results, many studies have attempted to prevent and treat delirium using pharmacological and non-pharmacological interventions [3,4,5].

Risk factors for delirium have been suggested to include older age; cognitive, functional, and sensory impairment; infection; illness severity; renal and electrolyte disturbances; living in an institution; diabetes; cerebral vascular diseases; pulmonary diseases; opioid use; length of surgery; blood loss; transfusion; albumin, hematocrit, and hemoglobin levels; Mini-Mental State Examination score; inability to ambulate, depression, number of medications, and treatment with multiple drugs [6,7]. Based on these risk factors, non-pharmacological interventions are being applied to reduce one or more risk factors to prevent or treat delirium [3].

Non-pharmacological interventions applied to reduce these risk factors have been studied as single [8,9,10,11] or multi-component interventions [12,13,14]. In addition, intervention providers have been diverse, including primary care nurses, geriatric internists, psychiatrists, cardiologists, caregivers, and family members [1,13,15].

However, among these studies, there were many studies in which nurses were not included as intervention providers in clinical settings [16,17]. In addition, even a study that conducted a systematic review focusing on nursing interventions was a narrative review including before and after cohort studies and non-randomized controlled trials (NRCT) studies conducted from 1999 to 2014 [18]. In addition, there was a narrative review for nursing interventions to prevent delirium in intensive care unit patients during the COVID-19 pandemic [19].

However, nurses may have difficulties in providing interventions according to the intervention protocols presented in these studies. This is because non-pharmacological interventions provided by doctors or other healthcare providers may be difficult to implement in clinical practice led by nurses. In addition, in the case of studies that are not randomized controlled trials (RCTs), there is a limit to reflecting study results in practice when providing evidence-based nursing care.

In this study, a systematic review of non-pharmacological nursing interventions for the prevention and treatment of delirium is expected to help nurses immediately select and utilize interventions to apply in clinical nursing practice. In addition, it is expected that nurses can lead a multidisciplinary group consisting of various healthcare providers and provide non-pharmacological interventions. The results of a systematic review of RCTs will identify approaches for high-quality nursing care and suggest directions for future nursing research.

## 2. Materials and Methods

In this study, a systematic review was performed according to the Preferential Reporting Items for Systematic Reviews and Meta-Analyses (PRISMA) guidelines (Supplementary File S1) [20] to identify suitable non-pharmacological nursing interventions for the prevention and treatment of delirium in hospitalized adult patients. This systematic review was registered with PROSPERO on 18 January 2021 (CRD42021226538).

### 2.1. Selection Criteria

#### 2.1.1. Inclusion Criteria

The key question considered in the systematic literature review was “What are the non-pharmacological nursing interventions for the prevention and treatment of delirium in hospitalized adult patients?” The study was conducted following the Populations, Intervention, Comparison, Outcome, and Study Design (PICOSD) structure. The inclusion criteria were as follows: the population (P) consisted of hospitalized (medical unit, surgical unit, and intensive care unit) adult patients over 18 years of age, the interventions (I) were non-pharmacological nursing interventions, the comparison (C) was with usual care, the outcome (O) was prevention (incidence of delirium) and treatment (severity of delirium, duration of delirium) of delirium, and study design (SD) was all prospective RCTs. We searched the literature for studies with human participants published up to 27 January 2021 and included studies published in all languages.

#### 2.1.2. Exclusion Criteria

The exclusion criteria were as follows: (1) healthy volunteer, alcohol dependence, palliative care, long-term care, nursing home, pediatric unit, child, dementia, polypharmacy, animal subject; (2) risk factors, screening; (3) studies that used pharmacological interventions, electroconvulsive therapy, pain management, or restraint; (4) cost-effectiveness, mortality rate; and (5) studies that were qualitative studies, case studies, focus group interviews, mixed-method studies, protocols, commentaries, conference abstracts without full-text articles, non-RCTs, systematic reviews and meta-analyses, guidelines, reviews, letters, abstracts, editorials, comments, or studies reporting insufficient data.

### 2.2. Search Strategy and Data Extraction Criteria

#### 2.2.1. Search Strategy

The search strategy for the systematic review was developed and conducted by a literature search expert librarian experienced in systematic reviews with input from the authors of this study. On 27 January 2021, the search was conducted using the following electronic databases: PubMed, Cumulative Index for Nursing Allied Health Literature (CINAHL), Embase (Elsevier platform), and Cochrane Central Register of Randomized Controlled Trials (Wiley platform). The search terms included delirium and non-pharmacological interventions. Search results were exported to EndNote^®^ X8 (Clarivate Analytics, Philadelphia, PA, USA), and duplicate articles were removed.

#### 2.2.2. Study Selection

Two researchers independently evaluated the search results; after reviewing the title and abstract, the selected studies underwent a full-text review. Disagreements between researchers were addressed through discussion and, if necessary, a third researcher’s evaluation.

#### 2.2.3. Data Extraction

The first researcher extracted data from the studies included in this research, and the second researcher confirmed the accuracy of the extraction. Disagreements between the two researchers were addressed through discussion. The data to be extracted from each selected study included the general characteristics of the study (first author, publication year, country), study design, study participants (sample size, department, age of participants, and prescreening), methods of intervention (contents of intervention, providers, timing), control condition, delirium screening (incidence and severity), and outcome of the study (outcome, time points of measurements, and results of delirium).

### 2.3. Quality Assessment

Version 2 of the Cochrane Collaboration’s risk-of-bias tool (ROB 2) [21] was used to assess the quality of the selected RCTs. For each selected study, two researchers extracted and confirmed information in five domains: randomization process, deviations from the intended interventions, missing outcome data, measurement of the outcome, and selection of the reported result. Based on the RoB 2 result, the full text of each article was identified as exhibiting “high risk,” “some concerns,” or “low risk.” Two reviewers independently evaluated the articles and discussed any differences to reach a consensus.

## 3. Results

### 3.1. Selected Studies

**Figure 1:**
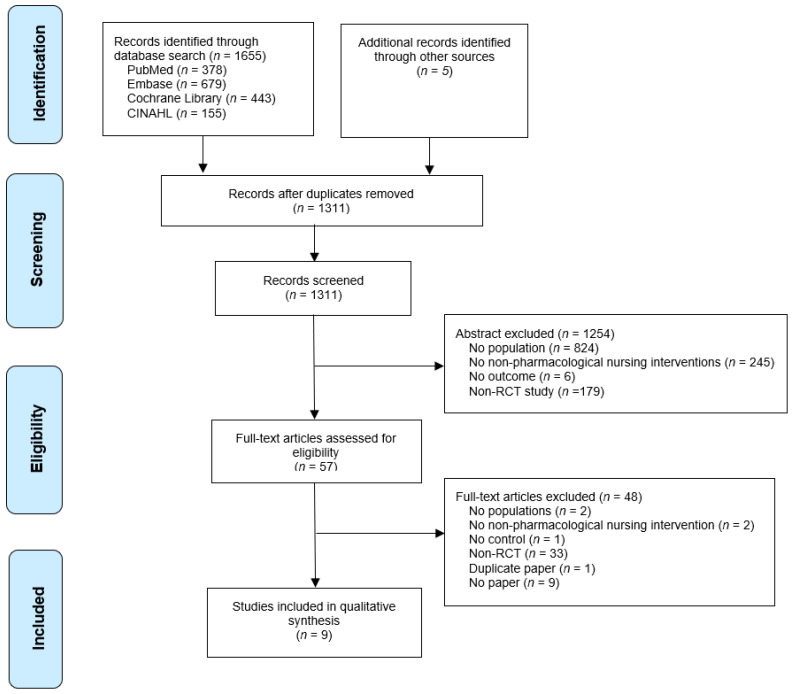
Preferred reporting items for systematic reviews (PRISMA) flow chart.

The study selection process for the systematic review is shown in Figure 1. As a result of searching the four databases, 1655 articles (378 in PubMed, 679 in Embase, 443 in the Cochrane Library, and 155 in CINAHL) were identified; a total of 1660 articles were identified by additionally searching five gray literature databases. After 349 duplicate studies were removed, the title and abstract of 1311 articles were checked, and 57 studies that met the inclusion criteria were selected. Fifty-seven full-text articles were reviewed, and two non-hospitalized population studies, two non-pharmacological nursing intervention studies, one study without a control group, 33 non-RCTs, one duplicate published study, and nine full-text articles were excluded. After excluding 48 articles, nine articles were finally included in the systematic review.

### 3.2. Study Characteristics

The characteristics of the nine studies included in this systematic review are summarized in Table 1. Six studies were published within the last five years [8,9,10,11,12,14] and three studies were published more than 5 years ago [13,22,23]. The studies were based in Belgium [23], Spain [12], Canada [10,13], Iran [8], China [14], the United States [9,11], and Japan [22]. There were a total of 877 participants across all studies, and the number of participants per study ranged from 10 to 112.

### 3.3. Risk of Bias Assessment

**Figure 2:**
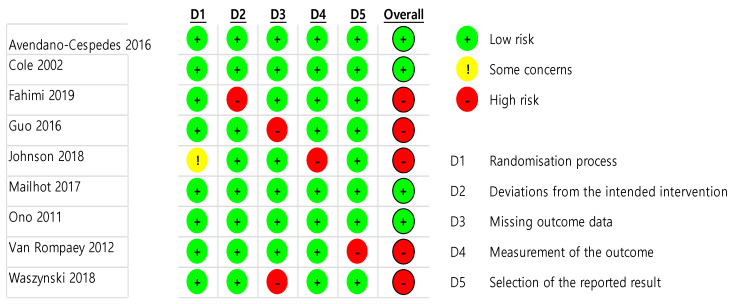
Risk of bias result.

**Figure 3:**
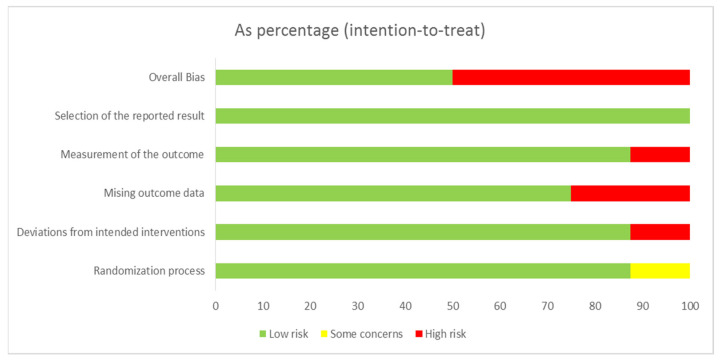
Risk of bias summary.

The quality assessment results of the eight selected studies are presented in Figure 2 and Figure 3. Regarding the overall bias, four studies had a low risk [10,12,13,22], and five studies had high risk [8,9,11,14,23]. Regarding the randomization process, eight studies had a low risk of bias [8,10,11,12,13,14,22,23], and one study had some concerns [9]. Regarding deviations from intended interventions, eight studies had a low risk of bias [9,10,11,12,13,14,22,23], and one study had a high risk [8]. Regarding missing outcome data, seven studies had a low risk of bias [8,9,10,12,13,22,23], and two studies had a high risk [11,14]. Regarding the measurement of the outcome, eight studies had a low risk of bias [8,10,11,12,13,14,22,23], and one study had a high risk [9]. Regarding the selection of the reported result, eight studies had a low risk of bias [8,9,10,11,12,13,14,22], and one study had a high risk [23].

### 3.4. Intervention and Outcome Measures

Analyzing the age of the study participants, three studies [12,13,14] included older adults aged over 65 years old, one study [9] included people over 55 years of age, and four studies [8,11,22,23] included adults over 18 years of age; one study [10] did not provide the age of participants.

The clinical units in which the study was conducted were the ward [8,12,13], intensive care unit (ICU) [14,23], and ward and ICU [9,10,22]; one study did not state any specific unit [11]. In addition, two studies [12,13] were conducted in a medical unit, six studies were conducted in a surgical unit [8,9,10,11,14,22], and one study [23] was conducted in medical and surgical units.

There were four studies of nurse-led interventions [10,11,12,23], and there were five studies in which nurses were included in various healthcare provider teams to provide the intervention [8,9,13,14,22].

There were four studies [8,12,13,14] on multi-component interventions and five studies on single-component interventions [9,10,11,22,23].

Regarding the prevention and treatment of delirium, four studies [8,9,22,23] examined delirium prevention, two studies [10,11] examined delirium treatment, and three studies [12,13,14] examined both prevention and treatment of delirium.

The types of non-pharmacological nursing interventions performed by nurses included multicomponent non-pharmacologic interventions [12,14], multidisciplinary care [13], multimedia education [8], music listening [9], mentoring of family caregivers concerning delirium management (MENTOR_D) [10], bright light exposure [22], ear plugs [23], and interventions for simulated family presence using pre-recorded video messages [11].

The contents of non-pharmacological nursing interventions included interventions including family members [10,11], multimedia interventions [11,13], music interventions [9,14], ear plugs [23], sleep management [12,14], orientation interventions [10,12,13,14], strengthened communication [13,14], and nutritional management [12,14].

Seven studies [8,9,10,11,12,13,14] included cognitive activities, and two studies [22,23] did not include cognitive activities.

Regarding the timing of providing the initial non-pharmacological nursing intervention, four studies were conducted from hospitalization [9,12,13,23], two studies were conducted from before surgery [8,14], one study was conducted from after surgery [22], and two studies [10,11] were conducted from after delirium occurred. Two studies [8,11] provided interventions only once, and seven studies [9,10,12,13,14,22,23] provided interventions periodically and repeatedly. The total time spent providing intervention was 1 min [11], 4–6 min [8], 150 min [10], 360 min [9], 480 min [22,23], 3 days [14], and 8 weeks [13]. One study continued providing the intervention from hospitalization to discharge [12].

Screening scales for delirium incidence were CAM [12], CAM-ICU [8,9,14], DSM-IV-TR [22], MMSE [13], and NEECHAM [23]. The scales used to measure the severity of delirium were the DRS [12], Delirium Index and CAM-ICU [10,13], and ABS [11].

The follow-up time in each intervention was 30 min [11], 3 days [9,10,14], 4 days [8], 5 days [22], 16 days [12], and up to 8 weeks [13]

As for the results of the application of non-pharmacological nursing interventions for the incidence of delirium, there were three studies [8,12,14] with a statistically significant difference, two studies [13,22] with no statistically significant difference, one study [9] did not develop delirium in both groups, and one study [23] did not suggest a statistically significant difference.

As a result of the application of non-pharmacological nursing interventions for the severity of delirium, there were two studies [11,12] with a statistically significant difference and two studies [10,13] without a statistically significant difference.

## 4. Discussion

This study aimed to examine the existing literature on non-pharmacological nursing interventions for the prevention and treatment of delirium in hospitalized adult patients. Further, it aimed to identify evidence that nurses could use in their clinical nursing practice and advance nursing research by conducting a systematic review.

Most previous studies that have confirmed the effectiveness of non-pharmacological interventions do not consider the providers of the interventions. Only a few studies were able to identify which interventions were performed by nurses; previous reviews related to non-pharmacological nursing interventions for the prevention and treatment of delirium in hospitalized adult patients included one integral review [24] and one narrative review [18].

In this study, a systematic literature review was conducted using only RCT-based studies. A recent systematic literature review of RCTs included a study to identify pharmacological and non-pharmacological interventions for the prevention and treatment of delirium after cardiac surgery [25], a study on the effects of family interventions in adults with delirium [15], a study that confirmed a non-pharmacological multicomponent intervention for the prevention of delirium in inpatients [1], and a study that confirmed the prevention and treatment of delirium in inpatients using physical training [26]. The above studies were conducted to confirm the effects of pharmacological interventions, or studies to confirm the effects of some interventions, such as family interventions and multi-component interventions; there were no studies to confirm the effects of non-pharmacological nursing interventions.

Non-pharmacological nursing interventions applied to prevent and treat delirium include multicomponent non-pharmacologic interventions, multidisciplinary care, multimedia education, music listening, mentoring of family caregivers concerning delirium management, bright light exposure, ear plugs, and interventions for simulated family presence using pre-recorded video messages. The results of previous studies confirmed non-pharmacological nursing interventions. One study confirmed the efficacy of a non-pharmacological intervention to prevent delirium in a general ward [3], and a narrative review [18] confirmed the efficacy of multicomponent programs as a nursing intervention to prevent delirium in hospitalized patients. Guidelines on the prevention and management of postoperative delirium in elderly patients [27] confirmed the efficacy of interventions using simulated family presence. However, there were many interventions that had different results from those in this study, and the types of interventions included in previous studies were inconsistent and varied. This study presented the theoretical basis for scientifically applying non-pharmacological nursing interventions in clinical practice, and suggests directions for nursing research.

That 66.7% (six) of studies were published within the last five years confirm that nurses’ interest in scientific interventions for the prevention and treatment of delirium has increased recently. In a previous study, delirium was reported to occur in 30–80% of hospitalized older adult patients, and the incidence did not decrease significantly over a long period of time [28,29]. This seems to reflect the efforts of nurses to apply various interventions to solve the problem because the incidence of delirium remains high despite continuous research by healthcare providers.

As a result of the quality assessment of the selected studies, the overall risk of bias was low in four studies (44.4%). This result was due to the high risk of bias of one or two studies in each of the five domains. In particular, two studies had a high risk of bias related to missing outcome data because of the large number of missing data after randomization. This is because older adult patients admitted to the intensive care unit often dropped out of the study following a change in the patient’s condition. Therefore, in the future, it is important to design studies that can account for missing data.

Nursing research for the prevention and treatment of delirium is being conducted worldwide, including in Europe, America, and Asia. This seems to reflect the high interest in identifying effective nursing interventions for delirium patients worldwide.

Nursing studies on delirium have been confirmed to have progressed the prevention and treatment of delirium. Multicomponent and multidisciplinary care has mainly been applied to the prevention and treatment of delirium. In addition, the provision of nursing care to the family has mainly applied to the treatment of delirium. It was confirmed that multimedia education, music listening, bright light exposure, and ear plugs were mainly applied in the prevention of delirium. It appears to be offered by a variety of healthcare providers, including a variety of interventions that apply to both the prevention and treatment of delirium. In addition, it seems that a single intervention was independently applied by nurses to prevent delirium.

The proportion of studies conducted in the intensive care unit and ward for the prevention and treatment of delirium was similar. Delirium is induced by various factors [17]; it was reported that 20% of older adults admitted to a medical ward have delirium [30], and delirium was the main factor underlying admission to the intensive care unit [31]. It was found that delirium in hospitalized adult patients was a nursing problem that had to be solved both in the ward and in the intensive care unit.

More studies on delirium were conducted in surgical departments (seven studies, 77%) than in medical departments. One study [30] found a higher incidence of delirium in patients who underwent cardiac and hip surgery; delirium appears to be recognized as a serious postoperative nursing problem that is being actively addressed.

In addition, the start of providing non-pharmacological nursing interventions for the prevention and treatment of delirium was applied from hospitalization in four (44%) of the selected studies. Since delirium is caused by various factors [17], it seems that interventions were preemptively implemented to prevent delirium after hospitalization.

More studies provided interventions repeatedly (7 studies, 77%) than studies that provided interventions only once. In a study [32] that confirmed the incidence of postoperative delirium by applying a music intervention to patients undergoing hip or knee surgery, it was confirmed that there were studies in which music intervention was repeatedly applied for at least 3 h a day for 3 days or more until discharge. Therefore, it appears that the most effective interventions require repeated delivery in future studies.

Single-component nursing intervention studies (five studies, 55%) were conducted slightly more often than multicomponent intervention studies. It is thought that nurses are trying to develop interventions in cooperation with various healthcare providers and are also trying to develop independent nursing interventions.

Seven studies (77%) included cognitive interventions. Since delirium manifests with symptoms of cognitive decline [25], interventions to maintain cognitive status, including cognitive activity, are mainly applied.

### Limitations

This study was conducted as a systematic review to identify non-pharmacological nursing interventions for the prevention and treatment of delirium in hospitalized adult patients; however, it has several limitations. First, it may be difficult to generalize the non-pharmacological interventions included in this study when applied to hospitalized adult patients because the intervention activities, providers, and timing of application were diverse. In this study, the gray literature search may have been insufficient, and although RCTs were targeted, there was a low overall risk of bias in only four out of nine studies. The screening scales for delirium incidence varied greatly between studies. In addition, since this study included a study in which nurses performed interventions as members of a research team, there is a limit to the analysis of non-pharmacological nursing interventions led by nurses.

## 5. Conclusions

Systematic reviews of non-pharmacological interventions for the prevention and treatment of delirium have been performed previously. Among these, studies on non-pharmacological nursing interventions were included; however, few studies were RCTs. This present study conducted a systematic review of the interventions, timing, and frequency of application of non-pharmacological nursing interventions for the prevention and treatment of delirium in RCTs. The contents of non-pharmacological nursing interventions include interventions including family members, multimedia interventions, music interventions, sleep management, orientation interventions, strengthened communication, and nutritional management. Nurses started providing interventions before hospitalization or surgery, and the time to apply the intervention varied from 1 min to the duration of the hospitalization, and most interventions were repeated several times. The results of this study can provide specific guidelines for nurses to select delirium nursing interventions that can be used in clinical practice. In addition, this study suggests directions for future studies on delirium nursing.

## Figures and Tables

**Table 1:** Descriptive summary of included studies.

	First Author, Publication Year, Country	Study Design	Patients	Intervention	Control Condition	Delirium Screening Scale	Outcome	Time points of Measurements	Delirium-Related Results
1	Avendaño-Céspedes 2016 Spain [12]	Parallel-group double-blind RCT	•50 hospitalized older adults Acute Geriatric Units •Exp: 21 patients Cont: 29 patients •Age: 65 years or older •No severe cognitive decline	**Intervention:** **Multicomponent non-pharmacologic intervention** (orientation, sensorial deficit, sleep, mobilization, hydration, nutrition, drug chart review, elimination, oxygenation, pain) **Provider:** intervention nurses **Timing:** Start—within 24 h of admission Duration—daily (from admission to discharge)	Usual care	**Incidence:** CAM **Severity**: Delirium Rating Scale-Revised-98 (DRS)	**Primary outcomes:** incidence, duration, severity **Secondary outcomes:** mortality, length of stay, use of physical restraint measures, and use of drugs for delirium control	**From admission to 16 days** Daily delirium evaluation in the afternoon	**Incidence** *p* = 0.039 Exp: 3 (14.3%) Cont: 12 (41.4%) **Severity** *p* = 0.040 Exp 35.0 (15.0%) Cont 65.0 (45.9%) **Duration** Exp: 1.7 (0.8) Cont: 3.4 (2.2)
2	Cole 2002 Canada	RCT	•218 older patients Five general medical units •Exp: 106 patients Cont: 112 patients •Age: 65 years or older •**Prescreening:** Short Portable Mental Status Questionnaire (scored 3–9 errors) or delirium recorded in the nursing notes + CAM	**Intervention:** **Multidisciplinary care** Consultation and follow up by a geriatric internist or psychiatrist and follow-up in hospital by the study nurse Nursing intervention protocol (environment, orientation, familiarity, communication, activity) **Provider:** intervention nurse, primary care nurses, geriatric internist, or psychiatrist **Timing:** Start—within 24 h of admission to detect prevalent delirium Duration—daily sessions with mean duration of 35.7 min for 8 weeks	Usual care	**Incidence:** Mini-Mental State Examination (MMSE) score **Severity:** Delirium Index CAM	**Primary outcomes:** incidence, severity	**Up to 8weeks** Three times during the first week and weekly thereafter for up to 8 weeks in hospital or until discharge	**Incidence** HR = 1.15, 95% CI 0.48–2.79 **Severity** HR = 1.09, 95% CI 0.74–1.60
3	Fahimi 2019 Iran	RCT	•110 patients undergoing a coronary artery bypass graft •Exp: 55 patients Cont: 55 patients •Age: 18 years or older •**Prescreening:** Richmond Agitation-Sedation Scale (RASS)	**Intervention:** **Multimedia education** multimedia CD containing three short educational videos of 4–6 min 1st video—provides information about the disease process and procedures for CABG 2nd video—describes postoperative measures and special care provided in the Department of Cardiac Surgery, patient visitation schedule and procedure, respiratory exercises, exercise for the foot that undergoes surgery and possible complications, and bed leave time 3rd video-pre- and postoperative experiences with the patient **Provider:** 1st video—cardiologist, 2nd video—heart surgical ICU nurse 3rd video—person who has already undergone CABG **Timing:** Start—5–7 d before surgery Duration—4–6 min	Usual care	**Incidence:** CAM-ICU	**Primary outcome:** Incidence	1~4 Postoperative day Twice a day(morning and afternoon) from admission to discharge from the ICU	**Incidence** Total *p* = 0.003 Exp: 13 (11.8) Cont: 28 (25.5) POD#2 morning *p* = 0.003 Exp: 4 (3.6) Cont: 16 (14.5) POD#3 morning *p* = 0.007 Exp: 0 (0) Cont: 8 (7.3) POD#4 morning *p* = 0.035 Exp: 0 (0) Cont: 6 (5.5)
4	Guo 2016 China	RCT	•160 elderly oral cancer patients who underwent tumor resection surgery Surgical intensive care unit •Exp: 81 patients Cont: 79 patients •Age: 65–80 years •**Prescreening:** MMSE score <24	**Intervention:** **Multicomponent, non-pharmacologic interventions: stimulating cognitive activities** Preoperative health education was strengthened and providing psychological guidance to the patients Invited the patients to visit the SICU to become acquainted with the environment Calendars, clocks, cell phones, radios, glasses, and hearing aids were repeatedly offered to accomplish time, place, and character orientation three times per day Effective communication using a communication card and WordPad Noise was decreased as much as possible Good sleep–wake cycle was adopted Between 23:00 and 05:00, all nursing procedures were minimized Eyeshade and acoustic earplugs were allocated No restraint straps or indwelling catheters were applied Bedside MP3 players were provided to play light music through headphones for 1 h three times daily Nasal feeding was administered as soon as possible Usual care also provided **Provider:** MNI team (including nurse) **Timing:** Start/duration—preoperation to SICU admission (total time: 3 d)	Usual care	**Incidence:** CAM-ICU QoR40 (40-item quality of recovery score)	**Primary outcomes:** incidence, duration **Secondary outcome:** melatonin sulfate	First three postoperative days Twice daily 07:00–08:00 (T1, T3, T5) 19:00–20:00 (T2,T4,T6)	**Incidence** Total *p* = 0.006 Exp: 10 (15%) Cont: 25 (31.6%) POD#1 *p* = 0.035 Exp: 4 (7.5%) Cont: 13 (16.25%) POD#2 *p* = 0.374 Exp: 5 (6.25%) Cont: 9 (11.25%) POD#3 *p* = 0.364 Exp: 1 (1.25%) Cont: 4 (5%) **Duration** *p* = 0.001 Exp: 28.1 (8.6) Cont: 60.2 (15.8)
5	Johnson 2018 United States	RCT	•40 patients Trauma Intensive Care and Trauma Orthopaedic Unit •Exp: 20 patients Cont: 20 patients •Age: 55 years or older •**Prescreening:** CAM-ICU negative on admission	**Intervention:** **Music listening** headphones and a numbered iPod shuffle preloaded with 60 min of pre-selected music (a) simple repetitive rhythm, (b) self-selection, and (c) slow tempo (60–80 BPM) **Provider:** nurses **Timing:** Start—following admission Duration—60 min, two times per day, at 14:00 and 20:00 over a 3-d period (total time: 360 min)	Usual care	**Incidence:** CAM-ICU	**Primary outcome:** incidence **Secondary outcome:** physiologic signs	From admission to three day Every twelve hours at the beginning of each shift; from 07:00 to 19:00 and 19:00 to 07:00	**Incidence** All participants screened negative for delirium.
6	Mailhot 2017 Canada	RCT	•30 patients Surgical intensive care unit (ICU) or the surgery unit •Exp: 16 patients Cont: 14 patients •**Prescreening:** Score ≥4 on the Intensive Care Delirium Screening Checklist (ICDSC)	**Intervention:** **Mentoring of family caregivers concerning delirium management (MENTOR_D)** Observe signs of delirium, communicate observations with the nurse, reorient patients, talk about family memories, use clear and simple sentences, verify if loved ones is wearing eyeglasses or hearing aids Usual care also provided **Provider:** intervention nurse (as a mentor who provided information on delirium and guidance to the FC in their new role of intervening in delirium management) **Timing:** Start—within 24 h of delirium onset with a total of seven encounters Duration—the first six encounters were 60 min, with 30 min for pre-bedside phase, 15 min for the bedside phase, and 15 min for post-bedside phase; and 30 min for the seventh discharge encounter (total time-150 min)	Usual care	**Severity:** Delirium Index (DI) CAM-ICU	**Primary outcomes:** severity, duration **Secondary outcomes:** complications during delirium, postoperative hospital stay, psycho-functional recovery, FC’s anxiety, self-efficacy	Days 1, 2, and 3 following study entry	**Severity** similar trajectories on days 1, 2, and 3 in both groups *p* = 0.27 Exp: Day 1: 10.56 (3.5%) Day 2: 5.38(5.45%) Day 3: 3.43 (4.96%) Cont: Day 1: 12.07 (4.05%) Day 2: 8 (6.34%) Day 3: 5.5 (7%) **Duration** Exp: 1.94 (1.34%) Cont: 4.14 (4.04%)
7	Ono 2011 Japan	RCT	•22 patients following esophagectomy ICU •Exp: 10 patients Cont:12 patients •Age: 18 years or older •**Prescreening:** NEECHAM Scale	**Intervention:** **Bright light exposure** Light at 2500 lx for the first 15 min (07:30–07:45), 4000 lx for the following 15 min (07:45–08:00), 5000 lx for 1 h (08:00–09:00), 4000 lx for 15 min (09:00–09:15) and 2500 lx for final 15 min (09:15–09:30) The light was a combination of daylight shining through the window, room lighting, and bright light exposure device. **Provider:** nurse **Timing** Start—postoperative day 2 Duration—2 h light exposure starting at 07:30 for 4 days (total time: 480 min)	Usual care	**Incidence:** DSM-IV-TR	**Primary outcome:** incidence **Secondary outcome:** rhythms in the activity and amount of movements at night as a proxy for sleeplessness, Heart rate variability and autonomic nervous system, and postoperative arrhythmia	From postoperative day 2 to postoperative day 5 Twice daily in the daytime and at night time	**Incidence** Exp: 1 (10%) Cont: 5 (42.7%) no significant difference
8	Van Rompaey 2012 Belgium	RCT	•136 patients ICU •Exp: 69 patients Cont: 67 patients •Age: 18 years or older •**Prescreening:** NEECHAM Scale	**Intervention:** **ear plugs** polyurethane Bilsom type **Provider:** assigned critical care nurse **Timing** 22:00–06:00	No action	**Incidence:** NEECHAM	**Primary outcome:** Incidence **Secondary outcome:** Sleep perception	From 0hours to 96hrs Maximum of five nights assessed during each nursing shift, at 08.00, 16.00, and 22.00	**Incidence** Exp: 20.3% Cont: 19.4%
9	Waszynski, 2018 United States	Single site RCT	•111 patients Acute care level one trauma center •Exp 1: 34 patients Exp 2: 41 patients Cont: 37 patients •Age: 18 years or older •**Prescreening:** Hospitalized patients experiencing hyperactive or mixed delirium and receiving continuous observation	**Intervention:** **simulated family presence using pre-recorded video messages** Watched a video on a DVD player placed on the over bed table located two feet in front of the participant Exp 1: view a 1-min family video message The message contained a personalized greeting delivered by one or more family members intended to provide a sense of calm and familiarity for the delirious participant Exp 2: view a 1-min nature videoA 1-min segment of a nature video containing images and sound of rain falling on colorful tropical plants or flowers was the attention control intervention Usual care also provided **Provider:** primary investigator (nurse) **Timing:** Start—administered the intervention immediately if the participant displayed any behaviors listed on the ABS Duration—1 min	Usual care	**Severity:** Agitated Behavior Scale	**Primary outcome:** agitation	immediately post intervention; 30-min post intervention Four time points (pre-intervention/baseline; during intervention; immediately post intervention; 30-min post intervention)	**Severity** Four time periods *p* < 0.001 Exp 1: 94.1% Cont: 29.7% Pre-intervention/baseline *p* = 0.071 Exp 1: 16 Exp 2: 17 Cont: 16 During intervention *p* < 0.001, d = 0.194 Exp 1: 14 Exp 2: 15 Cont: 16 Immediately post intervention *p* = 0.158 Exp 1: 14 Exp 2: 16 Cont: 16 30 min post intervention *p* = 0.971 Exp 1: 15 Exp 2: 15 Cont: 15

## Data Availability

The data that support the findings of this study are available from the corresponding author upon reasonable request.

## Supplementary Materials

The following are available online at https://www.mdpi.com/article/10.3390/ijerph18168853/s1, Material S1: PRISMA 2009 checklist.
